# Anatomia da trifurcação do tronco superior do plexo braquial: Nervo supraescapular, divisão posterior e divisão anterior

**DOI:** 10.1055/s-0045-1814117

**Published:** 2025-12-30

**Authors:** Junot Hortêncio de Souza Neto, Bernardo Couto Neto, André Bastos Duarte Eiras, Renato Harley Santos Botelho, Lucas Gonçalves Daflon, Marco Aurélio Rodrigues da Fonseca Passos

**Affiliations:** 1 Departamento de Cirurgia da Mão e Microcirurgia, Hospital Universitário Pedro Ernesto, Universidade do Estado do Rio de Janeiro, Rio de Janeiro, RJ, Brasil; 2 Departamento de Cirurgia da Mão, Hospital Naval Marcílio Dias, Marinha do Brasil, Rio de Janeiro, RJ, Brasil; 3 Departamento de Ortopedia e Traumatologia, Faculdade de Ciências Médicas, Universidade do Estado do Rio de Janeiro, Rio de Janeiro, RJ, Brasil; 4 Departamento de Ortopedia e Traumatologia, Faculdade de Medicina, Universidade Federal Fluminense, Niterói, RJ, Brasil; 5 Departamento de Cirurgia da Mão e Microcirurgia, Faculdade de Ciências Médicas, Universidade do Estado do Rio de Janeiro, Rio de Janeiro, RJ, Brasil; 6 Departamento de Cirurgia da Mão, Instituto Nacional de Traumatologia e Ortopedia, Rio de Janeiro, RJ, Brasil; 7 Departamento de Anatomia, Faculdade de Ciências Médicas, Universidade do Estado do Rio de Janeiro, Rio de Janeiro, RJ, Brasil

**Keywords:** adulto, cadáver, clavícula, plexo braquial, adult, brachial plexus, cadaver, clavicle

## Abstract

**Objetivo:**

Fornecer uma descrição abrangente da anatomia do tronco superior e de sua trifurcação distal por meio da dissecção de plexos braquiais obtidos de cadáveres adultos.

**Métodos:**

Foram dissecados 40 plexos braquiais de cadáveres adultos preservados por meio de uma técnica única, baseada em formalina, desenvolvida e utilizada pelo Departamento de Anatomia de nossa instituição. A dissecção bilateral foi realizada em 20 cadáveres, colocados em decúbito dorsal, com adução precisa do braço. Apesar de o foco principal ser a anatomia do tronco superior, uma exploração completa de todo o plexo braquial foi realizada mediante uma incisão prolongada.

**Resultados:**

A divisão posterior do tronco superior situou-se consistentemente entre o nervo supraescapular e a divisão anterior. Em 22 (55%) dos plexos braquiais dissecados, o nervo supraescapular originava-se da região proximal da divisão posterior do tronco superior, imediatamente após a bifurcação. Entre os 18 (45%) restantes, o nervo supraescapular originava-se diretamente do tronco superior.

**Conclusão:**

A trifurcação distal do tronco superior do plexo braquial inclui o nervo supraescapular e as divisões posterior e anterior do tronco superior.

## Abstract (en)

**Objective:**

To provide a comprehensive depiction of the anatomy of the upper trunk and its distal trifurcation through the dissection of brachial plexuses obtained from adult cadavers.

**Methods:**

We dissected 40 brachial plexuses from adult cadavers preserved using a unique formalin-based technique developed and used by the Anatomy Department of our institution. Bilateral dissection was performed on 20 cadavers placed in the dorsal decubitus position with accurate arm adduction. While the primary focus was on the upper trunk's anatomy, a thorough exploration of the entire brachial plexus was achieved through an extended incision.

**Results:**

The posterior division of the upper trunk was consistently located between the suprascapular nerve and the anterior division. In 22 (55%) of the dissected brachial plexuses, the suprascapular nerve originated from the proximal region of the posterior division of the upper trunk, immediately after its bifurcation. For the remaining 18 (45%), the suprascapular nerve originated directly from the upper trunk.

**Conclusion:**

The distal trifurcation of the upper trunk of the brachial plexus includes the suprascapular nerve and the posterior and anterior divisions of the upper trunk.

## Introdução

O triângulo interescalênico é uma passagem anatômica para a artéria subclávia e o plexo braquial, ao passo que a veia subclávia segue um curso anterior, fora desse espaço triangular. A veia é envolvida por uma bainha fascial, composta por porções das fáscias cervical profunda e clavipectoral. 1 Originário do ramo ventral dos nervos espinhais cervicais inferiores e do primeiro nervo espinhal torácico, o plexo braquial emerge da medula espinhal por meio das raízes e dos filamentos radiculares ventrais e dorsais. Ao sair do forame intervertebral, o nervo espinhal gera um ramo meníngeo recorrente, conhecido como *ramo de Luschka* , juntamente com um ramo dorsal responsável pela inervação sensorial e motora da região posterior do pescoço. Além disso, um ramo ventral contribui para a formação do plexo braquial. 2 3 4

O plexo braquial pode receber contribuições do quarto nervo espinhal cervical (C4; prefixado) ou do segundo nervo espinhal torácico (T2; pós-fixado). A literatura indica uma variabilidade considerável na prevalência de contribuições de C4 ou T2, com 67 a 75% consideradas típicas em plexos braquiais. Entre essas, 17,5 a 48% são classificadas como *prefixadas* , e 2 a 7,5%, como *pós-fixadas* . 5 6 7 8 O tronco superior forma-se pela fusão das raízes C5 a C6; o tronco médio consiste exclusivamente na raiz C7; e o tronco inferior resulta da união das raízes C8 a T1. Cada tronco dá origem a divisões anteriores e posteriores. As divisões posteriores convergem para formar o cordão posterior. Em contraste, as divisões anteriores dos troncos superior e médio se combinam para formar o cordão lateral, ao passo que a divisão anterior do tronco inferior continua a desenvolver o cordão medial. 9 10 11 Em casos de paralisia do tronco superior ou de lesões traumáticas, uma abordagem reconstrutiva potencial envolve utilizar a única raiz disponível para reinervar o deltoide por meio da divisão posterior do tronco superior. Transferências nervosas podem ser empregadas para restaurar a flexão do cotovelo. No entanto, a colocação incorreta de enxertos nervosos da única raiz disponível na divisão anterior do tronco superior e a utilização de transferências nervosas distais para a flexão do cotovelo podem levar à reinervação subótima do deltoide, devido à ausência de axônios intraplexuais na divisão posterior. Entender isso é crucial para evitar enxertar na divisão errada. 12

**Fig. 1:**
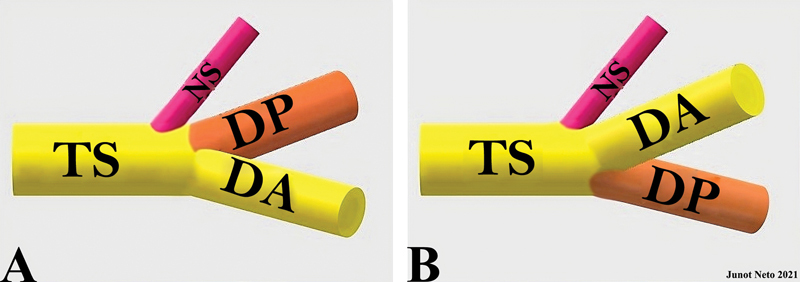
Segmento superior do plexo braquial. ( **A** ) Anatomia do tronco superior observada em dissecções de cadáveres. ( **B** ) Anatomia do tronco superior comumente apresentada em diversas fontes da literatura. **Abreviaturas:** TS, tronco superior; NS, nervo supraescapular; DP, divisão posterior; DA, divisão anterior.

Descrever a anatomia do plexo braquial continua sendo uma tarefa formidável, dada a sua complexidade intrínseca e a prevalência de variações anatômicas. 3 9 Em 1904, o Dr. Wilfred Harris, professor pioneiro no Departamento de Neurologia inaugural de um hospital universitário, escreveu um artigo intitulado “The True Form of the Brachial Plexus, and its Motor Distribution” (“A verdadeira forma do plexo braquial e sua distribuição motora”). 13 O O Dr. Harris argumentou que a divisão posterior do tronco superior do plexo braquial se originava consistentemente entre o nervo supraescapular e a divisão anterior do plexo braquial ( Fig. 1A ), ao contrário da maioria das representações e descrições contemporâneas ( Fig. 1B ). Fig. 1B ). 12 14 15 16 A incongruência na compreensão anatômica nos levou a realizar este estudo de dissecção de cadáveres para elucidar a anatomia do tronco superior do plexo braquial e suas divisões.

## Materiais e Métodos

Este estudo abrangeu a dissecção de 40 plexos braquiais de cadáveres adultos preservados por meio de uma técnica à base de formalina leve, desenvolvida e empregada pelo Departamento de Anatomia da nossa instituição. Realizou-se um exame bilateral em 20 casos, todos de indivíduos do sexo masculino, com idade entre 30 e 50 anos. Os cadáveres que apresentavam sinais de lesões ou suspeita de danos no plexo braquial foram intencionalmente excluídos do estudo. Felizmente, nenhum dos cadáveres dissecados exibiu lesões aparentes do plexo braquial.

As dissecções foram realizadas para elucidar as origens das divisões anterior e posterior do tronco superior do plexo braquial e sua relação com o nervo supraescapular. Os cadáveres foram posicionados em decúbito dorsal, com o braço aduzido, para manter a orientação anatômica adequada. Apesar de o foco principal ser a anatomia do tronco superior e de suas divisões, uma exposição abrangente de todo o plexo braquial em todos os cadáveres analisados foi alcançada por meio de uma incisão prolongada. Isso envolveu uma abordagem supraclavicular longitudinal ao longo da margem esternomastoidea, suplementada por uma incisão supraclavicular transversal sobre a clavícula, que se estendia inferiormente ao longo do sulco deltopeitoral.

A dissecção do plexo supraclavicular começou com uma incisão profunda na pele, que dividia o músculo platisma. Posteriormente, os nervos supraclaviculares foram identificados abaixo do músculo platisma, e a fáscia profunda foi incisada ao longo da linha especificada. Em alguns casos, as fibras da cabeça clavicular do músculo esternomastoideo foram dissecadas distalmente. Após essa etapa, o músculo omo-hióideo foi dividido, e a camada fascial que cobre o músculo escaleno foi incisada para expor o plexo braquial. Uma incisão foi meticulosamente feita através da pele, do tecido subcutâneo e da fáscia clavipectoral para acessar o plexo infraclavicular, estendendo-se até a clavícula. Posteriormente, o tendão do músculo peitoral menor foi localizado, elevado e incisado para revelar o comprimento completo do plexo infraclavicular. Em todos os casos, a osteotomia da clavícula foi realizada para otimizar a exposição. A anatomia do tronco superior do plexo braquial em todos os cadáveres dissecados foi completamente documentada por meio de fotografias, ilustrações e descrições escritas abrangentes. Esses registros foram compilados para análise posterior.

O estudo foi aprovado pelo comitê de ética da Plataforma Brasil sob o número CAAE 95856618.6.0000.5259.

## Resultados

**Fig. 2:**
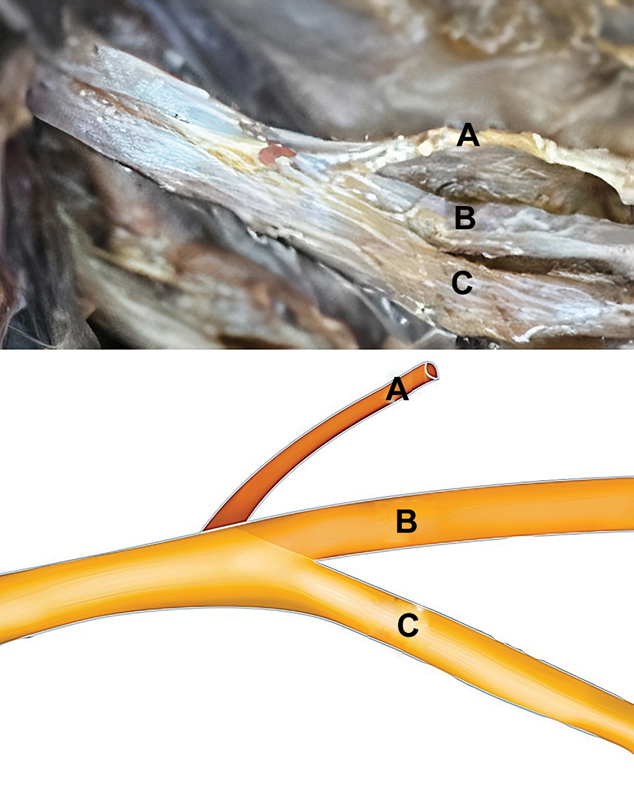
Anatomia do tronco superior baseada em dissecções em cadáveres. ( **A** ) O nervo supraescapular está posicionado mais cranialmente. ( **B** ) A divisão posterior tem origem cranial e dorsal, situada entre o nervo supraescapular e a divisão anterior do tronco superior. ( **C** ) A divisão anterior é a estrutura mais caudal com origem caudal e ventral.

Um padrão anatômico consistente foi observado em todos os 40 plexos braquiais dissecados dos 20 cadáveres. A divisão posterior do tronco superior ocupava consistentemente a posição mais craniana, e originava-se cranial e dorsalmente logo abaixo do nervo supraescapular. Em seguida, a divisão anterior do tronco superior assumiu a posição mais caudal no tronco superior do plexo braquial, com origens caudais e ventrais. Notavelmente, a divisão posterior do tronco superior situava-se consistentemente entre o nervo supraescapular e a divisão anterior ( Fig. 2 ).

Além disso, a origem do nervo supraescapular apresentou dois padrões distintos. Em 22 dos 40 plexos braquiais dissecados (55%), o nervo supraescapular originava-se da região proximal da divisão posterior do tronco superior, imediatamente após a bifurcação. Por outro lado, nos 18 casos restantes (45%), o nervo supraescapular originava-se diretamente do tronco superior.

## Discussão

Descrever o plexo braquial humano, por meio de texto ou ilustrações é um desafio persistente, apesar de séculos de estudos anatômicos. As dificuldades decorrem das interconexões complexas no plexo e da prevalência documentada de variações anatômicas na literatura. 3 9 Muitos autores reproduzem ilustrações familiares do plexo braquial sem examinar minuciosamente as relações espaciais de suas estruturas constituintes. Tais descrições frequentemente se limitam a interconexões reconhecidas, e assumem que essas representações fornecem uma estrutura suficiente para compreender essas conexões. Hanna 14 ressalta que, por simplicidade ou conveniência, o plexo braquial é frequentemente ilustrado com a divisão anterior do tronco superior posicionada mais cranialmente do que a divisão posterior ( Fig. 1B ). Hanna 14 sugere que esta convenção remonta provavelmente a Andreas Vesalius, em 1555, e reconhece que esse retrato pode perpetuar um mito, uma vez que, na prática, muitos cirurgiões de nervos periféricos sabem que a combinação precisa não é comumente divulgada na maioria dos livros e artigos científicos ( Fig. 1B ).

Leonardo da Vinci, principalmente conhecido como pintor, fez contribuições significativas para a anatomia humana no século XVI. Sob a orientação de Andrea del Verrocchio, Da Vinci embarcou em empreendimentos anatômicos que envolviam a dissecação de cadáveres e a criação de inúmeras notas e ilustrações. A proficiência de Da Vinci em dissecação, renderizações artísticas meticulosas e percepções anatômicas notáveis lhe renderam aclamação. Entre suas realizações notáveis estavam a dissecção e a representação do plexo braquial. Curiosamente, as ilustrações de Da Vinci retratam a divisão posterior do tronco superior com uma origem craniana, ao passo que a divisão anterior do tronco superior tem uma origem caudal 17 ( Fig. 1A ).

O reconhecimento inicial de uma descrição imprecisa da anatomia do plexo braquial pode ser rastreado até Harris, 13 em 1904, que concluiu que a divisão posterior do tronco superior tinha origem craniana em relação à divisão anterior. Ele afirmou que a divisão posterior residia consistentemente entre o nervo supraescapular e a divisão anterior ( Fig. 1A ), e enfatizou um desvio da anatomia comumente publicada nos livros e artigos de sua época ( Fig. 1B ). Em 2016, Hanna 14 também afirmou isso, ao destacar a trifurcação distal do tronco superior do plexo braquial, disposta do cranial ao caudal como o nervo supraescapular, e as divisões posterior e anterior. Hanna 14 denominou essa combinação de *combinação SPA* , em que “S” denota o nervo supraescapular, “P” representa a divisão posterior, e “A”, a divisão anterior ( Fig. 1A ).

**Fig. 3:**
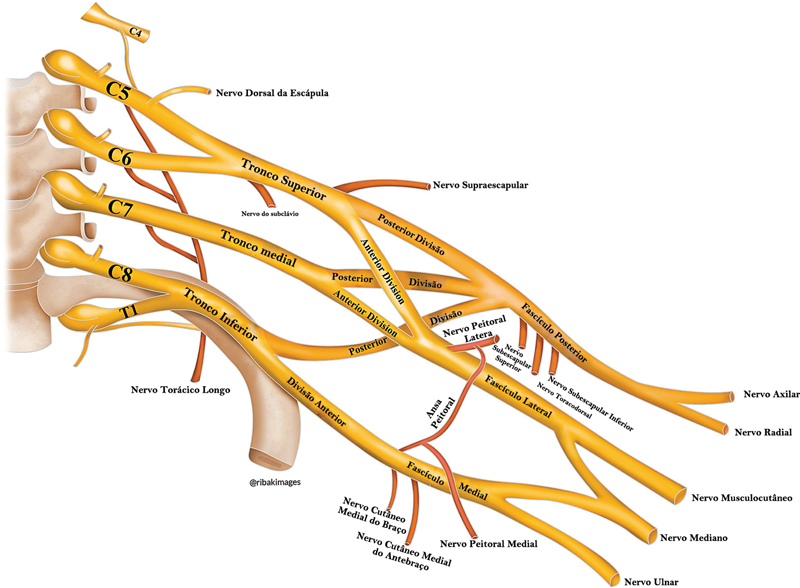
Ilustração do plexo braquial conforme os achados propostos por Neto et al. 15

Em 2015, Leung et al. 12 sugeriram que o trauma pode afetar a combinação das divisões no tronco superior. Para investigar isso, eles dissecaram 16 plexos de cadáveres frescos. Ao contrário de sua hipótese inicial, os autores 12 descobriram que o nervo supraescapular ocupava consistentemente a posição mais lateral no tronco superior. Após o nervo supraescapular, as divisões posterior e anterior formavam uma trifurcação, com a divisão posterior posicionada consistentemente entre o nervo supraescapular e a divisão anterior ( Fig. 1A ). Neto et al. 15 chegaram a uma conclusão semelhante, ao observar que o tronco superior do plexo braquial apresentava uma trifurcação distal. Do crânio ao caudal, o tronco superior incluía o nervo supraescapular e suas divisões posterior e anterior. Mais uma vez, a divisão posterior ocupava consistentemente o espaço entre o nervo supraescapular e a divisão anterior ( Fig. 1A ). Neto et al. 15 ilustraram um plexo braquial semelhante ao apresentado na Fig. 3 .

Arad et al. 16 relataram que, em 61% dos plexos braquiais dissecados, o nervo supraescapular originava-se da divisão posterior do tronco superior. Em comparação, em 35% dos casos, originava-se diretamente do tronco superior. Apenas 4% dos casos apresentaram o nervo supraescapular originário da raiz C5. Nossos achados revelaram que o nervo supraescapular emergia da divisão posterior imediatamente após sua origem em 22 casos (55%), ao passo que em 18 (45%), originava-se diretamente do tronco superior. Nenhum dos plexos dissecados apresentava o nervo supraescapular proveniente da raiz C5. Assim como Arad et al. 16 , concordamos que determinar a origem precisa do nervo supraescapular pode ser um desafio devido à sua proximidade com o ponto de bifurcação do tronco superior e às múltiplas camadas de mesoneuro que o circundam. É importante ressaltar que, quando Arad et al. 16 concluíram que o nervo supraescapular comumente se originava da divisão posterior, eles reconheceram que a divisão posterior do tronco superior do plexo braquial tinha origem craniana em comparação com sua divisão anterior ( Fig. 1A ). Essa observação ressalta que o tronco superior do plexo braquial e seus ramos se manifestam na cirurgia de forma diferente da que é tipicamente descrita na maioria dos livros de anatomia.

## Conclusão

O tronco superior do plexo braquial exibe uma trifurcação distal consistente e distinta, conhecida como a *combinação SPA* , conforme descrita por Hanna. 14 Nesta combinação, do cranial ao caudal, o tronco superior inclui o nervo supraescapular e as divisões posterior e anterior do tronco superior. Na avaliação, em 22 (55%) casos, o nervo supraescapular originava-se da divisão posterior do tronco superior, imediatamente após a sua origem. Nos 18 (45%) casos restantes, o nervo supraescapular originava-se diretamente do próprio tronco superior.
